# Influence of Maturity Status and Gender on Body Composition and Physical Fitness Adaptations to a Mobile-App-Based Physical Activity Intervention in Adolescents

**DOI:** 10.3390/sports14080317

**Published:** 2026-07-24

**Authors:** Victoria López-Lombó, Adrián Mateo-Orcajada, Lucía Abenza-Cano, J. Arturo Abraldes, Mario Albaladejo-Saura, Raquel Vaquero-Cristóbal

**Affiliations:** 1 Research Group Movement Sciences and Sport (MS&SPORT), Department of Physical Activity and Sport Sciences, Faculty of Sport Sciences, University of Murcia, 30720 San Javier, Murcia, Spain; victoria.lopezl@um.es (V.L.-L.); abraldes@um.es (J.A.A.); raquel.vaquero@um.es (R.V.-C.); 2 Facultad de Deporte, UCAM Universidad Católica de Murcia, 30107 Guadalupe, Murcia, Spain; labenza@ucam.edu (L.A.-C.); mdalbaladejosaura@ucam.edu (M.A.-S.)

**Keywords:** adolescents, body composition, gender, maturity, mobile applications, physical fitness

## Abstract

The use of mobile applications to promote physical activity in adolescents has shown promising results regarding body composition and fitness. However, current evidence is limited by treating adolescents as a homogeneous group, often overlooking whether biological maturity status modulates these effects. This randomized controlled trial analyzed whether biological maturity status and gender modulate the effects of a 10-week step-tracker app intervention in 395 adolescents (13.95 ± 1.20 years). The experimental group (EG) completed walking sessions at least three times per week recorded via the app, while the control group (CG) maintained habitual activities. Somatic maturation was estimated via predicted age at peak height velocity. The 10-week intervention consisted of an app-based step-tracker program (≥3 sessions/week, with goals scaling up to 12,500 steps day). Biological maturity status does not act as a global moderator of the intervention, with its influence being strictly restricted to minor, outcome-specific variations in adiposity metrics among early-maturing males. Males in the EG achieved significant reductions in fat mass (*p* = 0.002; $\eta_{p}^{2}$ = 0.025) and skinfold thickness (*p* = 0.005; $\eta_{p}^{2}$ = 0.021) compared to the CG. While physical fitness gains in adolescent males across both groups were predominantly driven by natural pubertal growth, females in the EG demonstrated specific intervention-induced improvements in abdominal muscular endurance (*p* = 0.001–0.042; $\eta_{p}^{2}$ = 0.011–0.028) and handgrip strength (*p* = 0.009–0.031; $\eta_{p}^{2}$ = 0.012–0.018) that were absent in controls. In conclusion, biological maturity status and gender show outcome-specific interactive effects on adolescent adiposity and physical fitness, though the associated effect sizes remain consistently small to trivial. Given these results, a standardized mobile step-tracking strategy remains a viable and inclusive tool for educational settings, while the design of gender-tailored digital frameworks should be treated cautiously as a hypothesis for future research.

## 1. Introduction

Adolescence is a crucial stage in human development [1]. During this period, healthy lifestyle habits are shaped that will be maintained for the rest of one’s life and will help prevent numerous chronic diseases [2]. However, recent research has shown that in recent years there has been an increase in rates of physical inactivity among adolescents, defined as less than sixty minutes of moderate to vigorous physical activity per day [3]. Furthermore, physical inactivity increases over the years during adolescence, affecting more than 80% of the population in the final years of this stage [3,4], becoming a public health problem [3]. Likewise, since the COVID-19 pandemic, changes have been observed in the use of free time in this population, which have led to a decline in physical activity and an increase in the use of new technologies [1].

Due to the increased use of new technologies among adolescents, and as a result of the reduction in hours of physical activity outside school hours [5,6,7], previous research has included mobile applications in intervention programs as a way to encourage physical activity in adolescents’ free time [1,8]. These studies have shown that mobile applications motivate adolescents to achieve their physical activity goals and challenges, preventing physical inactivity and sedentary lifestyles [8,9]. In addition, these changes in physical activity levels promote changes in body composition and physical fitness toward healthier parameters [10,11].

Therefore, interventions that promote physical activity using mobile applications appear to be useful for achieving health benefits in adolescents, although certain aspects must be taken into consideration [12]. First, the effectiveness of the interventions differs according to gender, with females making greater use of these mobile applications [12,13]. Second, the academic year has also been shown to be a determining factor, with adolescents in higher grades making a greater use of these devices [14]. Third, the previous level of physical activity of adolescents seems to influence the benefits obtained after the intervention, with inactive adolescents perceiving an increase in their physical activity level, as well as improvements in the study variables [15]. And fourth, the weight status of adolescents seems to be relevant, as those who are overweight or obese do not achieve such significant benefits after the intervention [16,17]. Collectively, these findings indicate that the effectiveness of mHealth interventions is not uniform across adolescents, suggesting that this population should not be viewed as a homogeneous group when evaluating the impact of such interventions.

Biological maturation may constitute another major source of heterogeneity. In this regard, hormonal changes occur during adolescence, leading to adaptations in body composition and physical fitness [12]. However, not all subjects mature at the same rate, and females tend to mature earlier than males [18]. Thus, adolescents who mature early show significantly higher values in anthropometric and derived variables, such as body mass, height, or muscle mass, and physical performance, especially in tests that depend on strength and power [19,20]. In contrast, adolescents who mature late have lower muscle mass and physical performance [21]. This could be because, prior to the age of peak height velocity (APHV), low levels of steroid hormones do not produce structural adaptations with physical exercise, with most adaptations being neuromuscular, while after the APHV there is a combination of neuromuscular and structural adaptations [22,23].

Beyond these purely physiological shifts, biological maturation and gender may also influence how adolescents interact with and adapt to mHealth behavioral tracking programs [13]. Structurally, because early maturers possess more advanced neuromuscular development and higher baseline physical capacities [19,20], identical digital challenges might impose a lower relative physiological strain on them compared to late maturers. This discrepancy in relative exercise intensity could directly influence daily physical fatigue, psychological enjoyment, and subsequent behavioral adherence to the digital tracking protocol [24,25], ultimately driving differential adaptations in body composition and fitness parameters.

Although previous international studies have identified several factors influencing the effectiveness of app-based physical activity interventions, including gender [12,13], school grade [13], baseline physical activity level [14], and weight status, biological maturation has been consistently overlooked. This represents an important limitation because adolescence is characterized by marked inter-individual variability in biological development that cannot be explained by chronological age alone [12]. Since maturation influences hormonal profile, body composition, neuromuscular development, and responsiveness to exercise training [26], adolescents with the same chronological age may therefore be expected to exhibit substantially different adaptations to identical physical activity interventions [27]. Understanding whether biological maturation influences intervention effectiveness would enable the design of more personalized mHealth strategies, improving exercise prescription during adolescence and optimizing health outcomes across different development stages. Consequently, interpreting intervention effects without considering maturity status may mask differential responses and partially explain the heterogeneous findings reported in previous mHealth interventions.

To date, few studies have examined how biological maturity status acts as an active moderator, rather than a simple baseline covariate, in mHealth physical activity interventions. This study presents a secondary analysis derived from a previously published research cohort. Crucially, while prior publications from this trial established general physical behavioral profiles, analyzed the distinct effects on active versus inactive adolescents, or treated gender and maturity status merely as static baseline covariates to control for pre-test confounding variance, the unique and novel value of the current analysis lies in exploring these variables as active independent moderators. That is, the study aims to identify how distinct developmental stages actively dictate the magnitude and direction of an adolescent’s functional adaptation to digital tracking through higher-order interaction modeling. Therefore, the present research aimed to achieve the following: (a) to determine whether biological maturity status moderates the effects of a 10-week mobile-app-based physical activity intervention on physical activity levels, body composition and physical fitness in adolescence; (b) to determinate the interaction of gender on the changes observed in the study variables.

Based on previous research, the hypotheses in the present study were that among early maturers, significantly greater pre-post changes will be directed toward anthropometric and derived variables and in physical fitness parameters that depend on muscle development, strength, and power compared to on-time and late maturers (H1); regarding gender interactions, the hypothesis is that females will show a significantly more pronounced directional improvement in body composition parameters, while males will show significantly greater relative improvements in physical fitness variables (H2), reflecting the divergent maturation rates and biological specializations characteristic of each sex. Given the lack of previous research on the interaction between maturation and physical activity programs promoted in the educational setting, it was not possible to establish a hypothesis regarding the changes in this variable with maturation.

## 2. Materials and Methods

### 2.1. Design

This study comprised a 10-week randomized controlled trial (RCT) flanked by 1-week pre- and post-test assessment periods, conducted between December 2022 and April 2023. Participants were allocated into either an experimental group (EG) or a control group (CG). The EG engaged in an after-school mobile application-based program (utilizing Strava, Pacer, MapMyWalk, and Pokémon Go) at least three times per week, with progressive weekly distance targets.

The primary outcome was the subjective physical activity level because the digital intervention targeted overall physical activity behavior via daily step goals. Secondary outcomes encompassed anthropometrics and body composition variables, and physical fitness variables. Biological maturity status and gender were established as the primary independent study moderators. This study presents a novel secondary analysis derived from an existing research dataset. Unlike prior publications from this trial that treated developmental variables as static baseline covariates to isolate behavioral profiles, the unique contribution of the current analysis lies in exploring biological maturation and gender as active independent moderators through higher-order interaction modeling.

The protocol adhered to the Declaration of Helsinki, received approval from the Institutional Ethics Committee of the Catholic University of Murcia (code: CE022102), was prospectively registered at ClinicalTrials.gov (code: NCT04860128) and followed the CONSORT guidelines.

### 2.2. Participants

A convenience sampling approach was utilized across two public secondary education facilities in the Region of Murcia (Murcia, Spain) (each boasting an enrollment over 200 students). Following administrative and institutional clearance, voluntary parental and participant signed informed consent was obtained, authorizing data collection and the anonymous publication of the results.

Based on prior literature utilizing digital step-trackers (SD = 0.60) [1], sample size calculations determined that a minimum of 380 adolescents was required to achieve a 95% confidence interval (95% CI) with an accepted margin of error of 0.06.

The inclusion criteria were age between 12 and 16 years old, absence of medical contraindications for physical activity, and enrollment in compulsory secondary education; while exclusion criteria were physical education class attendance < 80%, school relocation during the trial, or modifications to external extracurricular exercise routines. EG participants also required a compatible smartphone.

**Figure 1:**
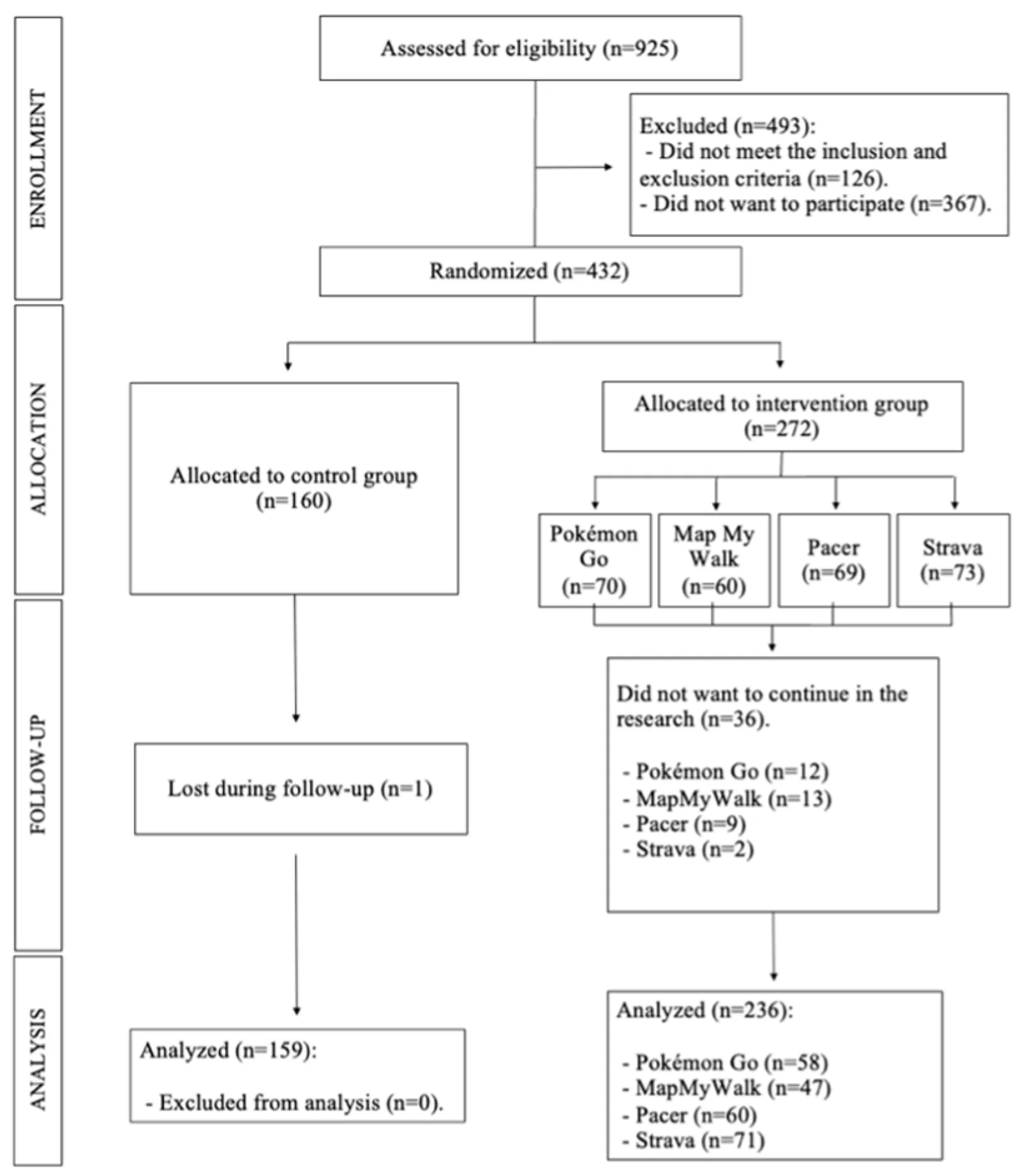
Sample selection flowchart.

From an initially eligible pool of 925 adolescents, 432 enrolled, and 395 successfully completed the trial (236 EG; 159 CG; 208 males and 187 females; mean age: 13.95 ± 1.20 years). The sample selection flowchart is shown in Figure 1.

### 2.3. Randomization and Blinding

The principal investigator in the presence of other researchers who were not involved in the study performed the allocation using a computer-generated random number table with a 1.5:1 ratio (EG to CG) to buffer against typical digital intervention dropout [1,28]. A secondary randomization distributed EG participants homogeneously among the four mobile applications (Strava, Pacer, MapMyWalk, and Pokémon Go).

To ensure robust double-blinding, data collectors and intervention supervisors remained completely unaware of group assignments, chronological test history, and participants’ maturation stages.

### 2.4. Instruments

The instruments used in this study were selected because they are valid and reliable tools for measuring adolescents.

#### 2.4.1. Questionnaires

The Physical Activity Questionnaire for Adolescents (PAQ-A) assessed physical activity levels via 9 items [29]. The final score was derived from the arithmetic mean of the first eight items [30], scored on a 1–5 Likert scale (ICC = 0.71) [31].

#### 2.4.2. Anthropometric and Body Composition Measurements

Assessments were conducted by International Society for the Advancement of Kinanthropometry (ISAK) accredited anthropometrists following standard protocols [32]. Metrics included basic measurements [body mass, using a TANITA BC 418-MA segmental scale (TANITA, Tokyo, Japan, accuracy: 100 g); height and sitting height, via SECA 213 stadiometer (SECA, Hamburg, Germany, accuracy: 0.1 cm)], three skinfolds [triceps, thigh and calf, via Harpenden caliper (Burgess Hill, United Kingdom, accuracy: 0.2 mm)], and five girths [relaxed arm, waist, hips, thigh, and calf, via Lufkin W606PM inextensible tape (Lufkin Industries, Missouri City, TX, USA, accuracy: 0.1 cm)]. All instruments were calibrated prior to the pre- and post-test measurements. In the pre and post measurements, each anthropometrist measured the same subjects to minimize inter-rater error.

These values computed BMI, muscle mass [33], fat mass [34], sum of 3 skinfolds, corrected arm girth (relaxed arm girth—[π × triceps skinfold]), corrected thigh girth (mid-thigh girth—[π × thigh skinfold]), corrected calf girth (calf girth—[π × calf skinfold], sum of corrected girths, and waist-to-height ratio (waist girth/height) [35].

Two measurements were taken for each variable, and a third measurement was taken when the difference was greater than 5% in skinfolds or 1% in the rest of the measurements. When two measurements were taken, the mean of the two was used, but when a third measurement was taken, the median of the three measurements was used [32].

Intra- and inter-evaluator technical measurement error (TEM) was calculated in a subsample. For the basic measurements, the intra- and inter-evaluator TEM was 0.03% and 0.05%, respectively; 1.23% and 1.99% for skinfolds, respectively; and 0.03% and 0.05% for girths, respectively.

#### 2.4.3. Biological Maturation

Somatic maturation was determined by predicting the maturity offset, which is defined as the estimated distance in years an adolescent is either before (yielding a negative value) or after (yielding a positive value) reaching peak height velocity (PHV) [36]. This parameter was calculated utilizing the sex-specific longitudinal equations proposed by Mirwald et al. [37], an approach that has demonstrated strong reliability in adolescent populations, including a high intraclass correlation coefficient (ICC = 0.96), a low coefficient of percentage variance (CV% = 0.8) and a minimal standard error (SE = 0.1) [38]. This method provides a non-invasive mathematical projection of somatic growth trajectories rather than implying that all participants have already achieved biological milestones, making it suitable for samples containing individuals in pre-, co-, and post-PHV states. The estimated APHV was subsequently derived as follows: APHV = chronological age–maturity offset.

In this sample, the overall mean APHV was 13.69 ± 0.62 years for males and 12.41 ± 0.56 for females. To optimize internal grouping validity and control for maturity-associated developmental differences, participants were stratified into three maturation cohorts using a ±0.5-year threshold relative to the sex-specific sample mean APHV, a conventional and widely adopted classification boundary in adolescent exercise science that effectively isolates distinct operational stages of biological acceleration [39]: early maturation (*n* = 63; <−0.5 years), on-time maturation (*n* = 185; ±0.5 years), and late maturation (*n* = 145; >+0.5 years). Characterized by sex and maturation group, the specific descriptive profiles for APHV were: 12.75 ± 0.46 years for early males, 13.66 ± 0.26 years for on time males, and 14.57 ± 0.30 years for late males; and for females, 11.60 ± 0.29 years for early maturers, 12.39 ± 0.27 years for on time maturers and 13.24 ± 0.31 years for late maturers.

#### 2.4.4. Physical Fitness Tests

Five specialized researchers administered a comprehensive fitness battery following a familiarization session. To avoid bias among evaluators, each test was assigned to a researcher. Cardiorespiratory capacity, upper limb strength, lower limb power, and abdominal muscle strength and endurance were measured, following the protocol of previous studies [40].

Cardiorespiratory capacity: The 20 m shuttle run test was executed until volitional exhaustion, predicting VO_2_ max via the Léger equation [41]. This is a maximum incremental test with high validity and reliability for use with adolescents [42]. The objective of the test is to cover a distance of 20 m as many times as possible before the sound signal is heard.

Upper limb strength: Two tests were performed. Handgrip strength test, which is considered a valid and reliable measurement in adolescents [43]. The adolescents were asked to apply maximum handgrip strength with their elbows fully extended [44]. A Takei Tkk5401 portable digital dynamometer (Takei Scientific Instruments, Tokyo, Japan) was used. All adolescents performed the test with both hands separately. The other test was the push-up. The adolescents had to perform as many push-ups as they could in one minute or until exhaustion. To perform the push-ups, the adolescents started lying in a prone position with their arms at their sides, elbows bent at 90°, and toes in contact with the floor. The number of final repetitions in which the elbows were fully extended and flexed during the execution was counted [45].

Lower limb power: the counter-movement jump (CMJ) was used. Flight height was recorded on a 200 Hz force platform (MuscleLab, Stathelle, Norway). They had to perform a 90° knee flexion followed by maximum speed extension to perform a jump and reach the maximum possible flight height. The hands had to remain on the hips throughout the flight phase, the knees and ankles had to be fully extended after takeoff and until landing, and the adolescents had to keep their backs completely straight [46].

Abdominal muscle strength and endurance: the curl-up test was used. The adolescents were placed in a supine position with their feet flat on the floor, knees bent at 90°, and arms crossed over their chest. From this position, they had to perform as many trunk flexions as possible until exhaustion or until the end of one minute. Repetitions were considered valid when the upper back was no longer in contact with the floor [47].

### 2.5. Procedure

To minimize interference from possible confounding variables, pre- and post-test measurements were taken under conditions that were as similar as possible. The PAQ-A questionnaire was administered in a quiet classroom, followed by anthropometric evaluations inside temperature-controlled locker rooms between 8:30 a.m. and 2:30 p.m., taking advantage of physical education classes so that each class group was measured at the same time in the pre- and post-tests to avoid diurnal bias [48,49]. Once completed, an explanation was given to the adolescents about the physical fitness test procedure, after which they were familiarized. Fitness testing occurred in an indoor sports hall following a standardized progressive warm-up. Fitness tests were performed twice (best trial retained) with 2 min intra-test and 5 min inter-test rest intervals. The order in which the tests (handgrip, push-up, CMJ, and curl-up) were performed was random for each adolescent. The 20 m shuttle run test was invariably executed last to prevent residual fatigue confounding other metrics.

The recommendations of the National Strength and Conditioning Association (NSCA) were followed to establish the physical fitness assessment protocol, based on the metabolic demands of each test, as well as the fatigue generated by the tests and the time required for Recovery [50].

### 2.6. Mobile Application Intervention

The baseline sample consisted of the CG (*n* = 160) and the EG (*n* = 272), the latter distributed across Pokémon Go^®^ (*n* = 70), MapMyWalk^®^ (*n* = 60), Pacer^®^ (*n* = 69), and Strava^®^ (*n* = 73). Following software profiling and operational briefings, the EG used their assigned app after school ≥3 times/week for 10 weeks. Daily physical activity targets expanded by 600 steps each week, scaling from 7100 steps/day (Week 1) up to 12,500 steps/day (Week 10). This distance was used in previous research with mobile step-tracking apps [51] and was set to reflect the level of physical activity required for adolescents to be considered active or very active, as well as to improve their cardiorespiratory fitness and overall health [52,53]. Distance was prescribed in kilometers (1 km = 1565 steps, approximately) [54]. Pokémon Go provides a gamified ecosystem [55,56], whereas Pacer, Strava, and MapMyWalk supply formal behavior change features [57]. The CG continued to attend physical education classes as normal and perform the physical activity they did before the start of the research but did not use any mobile step-tracking applications.

Although objective tracking verified compliance during sessions across the four mobile applications used (Pokémon Go, MapMyWalk, Pacer, and Strava), software and structural disparities between these platforms precluded the aggregation of a uniform, standardized raw digital metric, thereby justifying the reliance on the validated PAQ-A for cross-group comparisons.

Regarding missing data management and intervention compliance, a modified intention-to-treat approach was adopted; adolescents who completed post-test assessments were retained in the final analysis regardless of their compliance with weekly app targets. Conversely, a complete-case analysis approach was applied strictly to those lost to follow-up. This approach was methodologically justified as the final overall study attrition rate was low (8.56%; 395 out of 432 completed), remaining well below the conservative 20% critical threshold widely accepted as the baseline for avoiding attrition bias in behavioral and mHealth randomized trials. Furthermore, preliminary analysis confirmed that baseline demographic and anthropometric characteristics did not significantly differ between dropouts and completers (*p* > 0.05), ensuring that the exclusion of missing cases did not introduce systematic selection bias.

Independent app-specific attrition rates were documented as follows: CG (0.60%), Strava (2.74%), Pacer (13.04%), Pokémon Go (17.14%) and MapMyWalk (21.67%).

Active participation remained high throughout the intervention: 96.06% from Week 1 through Week 4; 93.29% from Week 5 through Week 8; and 91.44% between Weeks 9 and 10. However, achievement of the progressive physical activity targets showed a linear decline as developmental demands intensified; weekly goal completion dropped from 96.46% in the first two weeks, to 81.77% in week 4, to 69.11% in Week 7, and to 54.68% in Week 10.

### 2.7. Statistical Analysis

The normality of the data was analyzed using the Kolmogorov–Smirnov test, as well as skewness and kurtosis. Since all variables followed a normal distribution, parametric statistics were employed. To analyze the effect of the intervention, a four-way repeated measures ANOVA (4-way mixed ANOVA) was conducted for each dependent variable. The model included the within-subjects factor Time (Pre vs. Post) and the between-subjects factors Group (EG vs. CG), Gender (Male vs. Female), and Maturational Status (Early, On-time, Late). The four-way interaction (Time × Group × Maturational Status × Gender) was examined to identify differential responses to the treatment. To strictly control the family-wise error rate across multiple simple effects and protect the model against the inflation of Type I errors due to the large number of multi-factor comparisons, a Bonferroni post hoc correction was applied across all outcome measures. Furthermore, to ensure that the specific mobile application utilized did not confound the results, a secondary mixed ANOVA was performed within the Experimental Group, including Time as the within-subjects factor and ‘App Type’ as the between-subjects factor. Effect size was estimated using partial eta squared ($\eta_{p}^{2}$), following conventional benchmarks: small ($\eta_{p}^{2}$ ≥ 0.01), medium ($\eta_{p}^{2}$ ≥ 0.06), and large ($\eta_{p}^{2}$ ≥ 0.14). A *p* value of *p* < 0.05 was used to establish statistical significance. The SPSS statistical package was used for the statistical analysis (Version.25.0; SPSS Inc., Chicago, IL, USA).

## 3. Results

### 3.1. Primary Interaction Effects: Maturity and Gender Moderation

The primary efficacy of the intervention was evaluated through higher-order interaction terms to determine how biological maturity and gender moderated the treatment outcomes (Table 1). The three-way (Time × Group × Maturity Status) interaction model revealed that somatic maturation stages did not significantly alter the intervention’s impact on the majority of the tracked variables (*p* > 0.221) (Supplementary Table S1). Notably, a significant moderating effect of maturity was exclusively observed in body fat dynamics, specifically for the sum of 3 skinfolds (*p* = 0.039; $\eta_{p}^{2}$ = 0.020) and total fat mass (*p* = 0.037; $\eta_{p}^{2}$ = 0.014) (Table 1).

When accounting for gender, the four-way (Time × Group × Maturity Status × Gender) (Table 1) interaction achieved statistical significance across several key physiological domains, including body mass (*p* = 0.046; $\eta_{p}^{2}$ = 0.001), height (*p* = 0.016; $\eta_{p}^{2}$ = 0.018), sum of 3 skinfolds (*p* = 0.016; $\eta_{p}^{2}$ = 0.012), waist girth (*p* = 0.049; $\eta_{p}^{2}$ = 0.012), fat mass (*p* = 0.014; $\eta_{p}^{2}\;$= 0.010), handgrip strength (*p* < 0.047; $\eta_{p}^{2}\;$= 0.007), and curl-up performance (*p* = 0.006; $\eta_{p}^{2}\;$= 0.013), underscoring that while these multi-factor interactions achieve statistical significance, their true effect sizes remain small. For the remaining variables, the higher-order interaction did not show a clear systematic pattern, with variations distributed across disparate maturity and gender sub-cohorts (Supplementary Table S1).

### 3.2. Gender Stratified Pre-Post Outcomes

The comprehensive within-group pre-to-post significance tests for both groups across all maturation stages for all variables have been compiled in the Supplementary Materials (Tables S2 and S3), providing a complete overview of both *p*-values and their corresponding effect sizes for every analyzed variable. This section describes only the significant and most relevant results.

As detailed in Supplementary Table S2, the male EG showed a significant pre-post increase in height across all maturation cohorts, with effect sizes ranging from small to moderate ($\eta_{p}^{2}\;$= 0.017 to 0.067). The largest intra-group variations within the male EG were concentrated in the on-time maturation cohort, which exhibited the highest effect sizes for body mass ($\eta_{p}^{2\;}$ = 0.098), VO_2_ max ($\eta_{p}^{2}\;$= 0.092), height ($\eta_{p}^{2}\;$= 0.067), muscle mass ($\eta_{p}^{2\;}$ = 0.056), and right arm handgrip strength ($\eta_{p}^{2}\;$= 0.048). Additionally, male early maturers in the EG showed a significant decrease in fat mass percentage ($\eta_{p}^{2}\;$= 0.025) and the sum of 3 skinfolds ($\eta_{p}^{2}\;$= 0.021). In the male control group (CG), significant pre-post increases were also concentrated in the on-time maturation cohort, particularly for height ($\eta_{p}^{2}\;$= 0.061), body mass ($\eta_{p}^{2\;}$ = 0.048), and hip girth ($\eta_{p}^{2\;}$ = 0.040).

As detailed in Supplementary Table S3, the female EG demonstrated significant pre-post increases across all three maturation cohorts for the sum of corrected girths ($\eta_{p}^{2}\;$= 0.015 to 0.050) and muscle mass ($\eta_{p}^{2}\;$= 0.013 to 0.050). The highest magnitudes of change within the female EG were consistently found in the on-time maturers, specifically for the sum of corrected girths ($\eta_{p}^{2}$ = 0.050), muscle mass ($\eta_{p}^{2}\;$= 0.050), corrected arm girth ($\eta_{p}^{2}\;$= 0.048), corrected thigh girth ($\eta_{p}^{2}\;$= 0.047), and hip girth ($\eta_{p}^{2}\;$= 0.045). In the female CG, significant pre-post increases occurred predominantly within the on-time maturation cohort, with the largest effect sizes noted for hip girth ($\eta_{p}^{2}\;$= 0.065) and body mass ($\eta_{p}^{2\;}$ = 0.040).

### 3.3. Mobile Application Interaction Effect

The effect of the “mobile application used” factor on the study outcomes was analyzed. The results showed no significant differences in any of the variables when considering the interaction (Time x App used): physical activity level (*p* = 0.417), body mass (*p* = 0.075), height (*p* = 0.744), BMI (*p* = 0.190), sum of three skinfolds (*p* = 0.266), corrected arm girth (*p* = 0.853), corrected thigh girth (*p* = 0.387), corrected calf girth (*p* = 0.197), sum of corrected girths (*p* = 0.404), waist girth (*p* = 0.070), hip girth (*p* = 0.175), waist-to-height ratio (*p* = 0.529), muscle mass (*p* = 0.544), fat mass (*p* = 0.568), VO_2_ max (*p* = 0.117), right arm handgrip (*p* = 0.500), left arm handgrip (*p* = 0.673), CMJ (*p* = 0.324), push-ups (*p* = 0.471), and curl-ups (*p* = 0.100). Therefore, there was no effect of the specific mobile application used on the changes achieved in the study variables.

## 4. Discussion

The primary objective of this study was to analyze whether biological maturity status moderates the adaptations generated by a 10-week mobile-app-based physical activity intervention in adolescents. The results showed that in both the EG and the CG, there was a significant increase in body mass, height, corrected arm girth, sum of corrected girths, hip girth, and muscle mass in all maturation groups, while handgrip strength and performance in push-ups and curl-ups increased in all maturation groups in the EG. These results are similar to those from previous studies conducted with adolescents, which found a significant increase in body composition variables related to body mass, height, and muscle mass in both the EG and CG [58,59], while physical performance only improved in the group that underwent the intervention [1]. This suggests that the intervention did not play a decisive role in overall anthropometric shifts; instead, these changes are primarily driven by natural pubertal growth. During this stage, marked increases in height and body mass occur due to characteristic hormonal surges [60]. Similarly, rising sex hormone concentrations [61,62] stimulate muscle mass accretion [63,64]. However, because direct endocrine, hormonal, or metabolic parameters were not assessed, these biological mechanisms must be interpreted strictly as plausible hypotheses rather than definitive conclusions.

However, the increase in handgrip and curl-up performance in all maturation groups in the EG could indicate that the intervention with mobile applications is beneficial for the physical fitness of adolescents, regardless of their stage of maturation. These results are similar to those from previous studies, in which adolescents who participated in the physical activity program using mobile applications showed improvements in the variables related to strength and power. One possible explanation for the improvement in curl-ups could be that the trunk muscles remain active while walking [65], which could benefit performance in this test after 10 weeks of intervention. However, the improvement in handgrip performance observed has no prior scientific evidence to support it, as some previous research had shown improvements in these variables after walking programs carried out with older adults [66]; while other research, mainly in young people, had shown no or minimal changes in these variables [67]. One possible explanation for these results is that the increase in physical activity achieved through walking might have led adolescents to generate neuromuscular adaptations derived from training, reporting gains in physical fitness tests that were not directly related to the exercise performed. These neuromuscular improvements have been observed in previous studies with active young people, although it is true that the intensity and type of walking performed were decisive in the improvements observed [68]. Nevertheless, given the lack of direct neuromuscular tracking in our design, these interpretations remain speculative, and future research is needed to analyze the reasons why improvements in upper body strength were achieved after this type of intervention, to study the type of walking or running performed by adolescents.

Another interesting finding regarding body composition was that corrected calf girth increased in the on-time maturers in the EG and CG, in the early maturers in the EG, and in the late maturers in the CG. The increase in corrected calf girth in adolescents following physical activity interventions is consistent with the findings reported in previous studies [1,51]. In this case, the maturation process seems to have a significant effect on this variable. Thus, in on-time maturers, the increase observed in both the CG and EG could be due to the effect of growth and maturation converging with the training program, which is consistent with the findings from previous studies [51,65], making it difficult to determine the contribution of each component to the change observed in this variable. On the other hand, the fact that improvements in the EG were only observed in early maturers could hypothetically indicate that the training program enhanced the muscle mass gains in adolescents who were already at an advanced stage of maturation, potentially due to greater anabolic sensitivity [69,70]. As for the gains observed only in late maturers in the CG, one possible explanation could be that the training did not have a sufficient impact on the leg muscles to generate a process of hypertrophy in adolescents whose stage of maturation was not yet optimal for producing gains in muscle mass, with the changes in this group being more related to neural adaptations [71,72]. However, further research is needed to analyze what other factors may be influencing the adaptations generated by this type of physical activity intervention depending on the stage of maturation of adolescents.

Regarding variables related to adiposity, only the EG showed a significant decrease in the sum of three skinfolds and fat mass, mainly in the early and late maturers group. The importance of these results lies in the fact that the intervention appears to have a significant effect on these variables, which is in line with previous research conducted with mobile applications, which found that the use of these devices led to a decrease in fat mass accumulation in adolescents [28]. The fact that on-time maturers did not see changes in these variables could be because early maturers are at a more advanced stage of hormonal development characterized by higher testosterone and estrogen production, which could promote increased muscle mass and adipose tissue mobilization [20,60], provided that it is accompanied by physical activity, as is the case with the proposed intervention. In the case of late maturers, the reason why this occurs does not seem to be clear in the previous scientific literature, although it appears that adolescents who mature later show a decrease in fat indicators with age, reaching young adulthood with less body fat [73,74]. Therefore, in variables related to adiposity, these findings suggest that the moderating role of biological maturation is outcome specific rather than general, being particularly evident in adiposity-related adaptations by the physical activity intervention.

It should be noted that changes in physical activity levels and VO_2_ max were only found in adolescents in the on-time and late groups. During adolescence, there is a decrease in physical activity among adolescents [56], which is more characteristic of adolescents who mature earlier, although the relationship between the influencing factors is complex [27]. Mobile-app-based interventions could be an effective alternative for adolescents who mature early, as a decrease in physical activity was observed in the CG, while an increase in this variable was observed in the EG. Although this increase was not statistically significant, it may have contributed to maintaining adequate levels of physical activity. Regarding the changes obtained in VO_2_ max, it should be noted that improvements were found in both the EG and the CG. This strongly reflects that the intervention does not play a decisive role in the cardiorespiratory capacity of adolescents, and that the variations observed are mainly a reflection of normal adolescent growth, since VO_2_ max begins to increase during early adolescence, reaching its peak at around 12 years of age in females and 14 years of age in males [75,76]. This could mean that the early maturers who participated in the intervention had already reached their peak VO_2_ max, or were close to reaching it, so the changes were more pronounced in the on-time and late maturers group, who had more room for improvement.

Based on the results obtained, the first research hypothesis, which stated that, among early-maturing adolescents, significant changes between the pre- and post-periods would focus more on anthropometric and derived variables, as well as on physical fitness parameters that depend on muscle development, strength, and power, compared to normally maturing and late-maturing adolescents, can be partially rejected. Although early maturers showed significant improvements in adiposity-related variables following the intervention, most anthropometric, body composition and physical fitness outcomes evolved similarly across maturation groups. In contrast, improvements in physical fitness parameters were mainly observed in the EG, but across all maturation stages, suggesting that the intervention exerted a positive effect on fitness outcomes independently of maturation status. Overall, these findings indicate significant improvements in adiposity-related variables following the intervention based on step-tracking applications during adolescence, although higher-order interaction effects were small to trivial, indicating that the overall influence of biological maturation on the intervention outcomes remains highly restricted and specific.

The second objective of the study was to determine the influence of gender on the changes observed in the study variables. Gender showed a significant influence on the variables of body composition and physical fitness in the EG and CG. In males in the EG and CG, a significant increase was observed in body mass, height, BMI, corrected girths, muscle mass, and hip girths, mainly in the on-time maturers, although also in the other maturation groups. In addition, significant reductions in fat mass and the sum of three skinfolds were observed in early maturers in the EG, but not in the CG. On their part, females in the EG and CG also showed an increase in body mass, height, corrected girths, hip girth, and muscle mass, mainly in the on-time group, although also in the early and late groups. However, no significant reductions in adiposity-related variables (fat mass and sum of 3 skinfolds) were observed in females. These results are similar to those from previous studies, in which the maturation process affected both males and females in the CG and EG, although with differences between the two genders in adiposity variables [77,78]. One plausible explanation for these results may be that during puberty, physical and hormonal changes occur that are different in males and females [61,79]. Thus, whereas males exhibit a greater musculoskeletal development [63,64], females tend to show a greater accumulation of adiposity [80,81]. The relevance of these results lies in the fact that training using mobile applications appears to be sufficient to obtain benefits in males, while it does not seem to be generating a sufficient effect in females to mitigate the increase in adiposity that occurs during adolescence. However, further research is needed to analyze the intensity of the training performed by males and females in order to discern whether this is a determining variable in the results found.

It should be noted that no differences were found between males and females in muscle development, as males in the EG showed significant increases in corrected arm and thigh girths, while females in the EG showed increases in the sum of corrected girths, as well as arm and thigh girths. Therefore, there was a significant increase in muscle mass in both genders, which is consistent with findings from other studies that reported comparable improvements in muscle mass in both genders after interventions with mobile applications [82,83]. However, these results are partially contrary to other studies that observed more marked differences between genders, although they also described a general trend toward increased muscle mass during adolescence [55,56]. One possible explanation for these results is that the hormones generated during puberty, mainly testosterone and estrogen, are steroids and have a considerable impact on muscle development in males and females [79,81,84]. In fact, it is likely that the effect of training with mobile applications on muscle mass was reduced because significant changes in these variables were also found in the CG, demonstrating that the maturation process perhaps had a greater impact on these changes than the training performed. Future research that continues to analyze the impact of maturity level on males and females participating in physical activity interventions with mobile applications is necessary in order to draw accurate conclusions about the benefits generated by these devices.

Highly relevant results were found in the area of physical fitness. This is because the males in the EG showed significant differences in variables such as VO_2_ max, handgrip, CMJ, curl-ups, and push-ups, but these differences were also present in the males in the CG, which highlights that these fitness gains are predominantly driven by natural growth and neuromuscular maturation. This attenuates the independent impact of the mobile app intervention in adolescent males. However, in females in the EG, significant differences were observed after the intervention in curl-ups in all maturation groups and in handgrip strength in on-time maturers, while in females in the CG there were no significant changes in these variables. The differences found in the physical fitness of females are consistent with previous studies that showed that mobile app interventions can improve muscle strength and physical fitness in adolescents, especially in females [15,85]. One possible explanation for these results is that females tend to decrease their physical activity more than males during adolescence [86,87], leading to a greater decline in their physical fitness during this stage [87]. The increase in physical activity levels shown in females after the intervention could have a sufficiently positive impact on physical fitness variables, regardless of the fact that the intensity and volume were not high. This has been demonstrated in previous studies in which increased physical activity in adolescent females led to changes in body composition and physical fitness, regardless of the intensity of the activity [1]. This leads us to believe that interventions using mobile apps are extremely useful in adolescent females for generating change, regardless of their stage of development.

Based on the results obtained, the second research hypothesis, which stated that females would show a significantly more pronounced directional improvement in body composition parameters, while males would show significantly greater relative improvements in physical fitness variables, can be rejected. The main differences between males and females were observed in the variables related to adiposity, where females did not show significant decreases after the intervention, while males did so; as well as in the physical fitness variables, where females in the EG perceived benefits compared to those in the CG, although this did not occur in the case of males. This reflects that the maturation process affects males and females differently, as males showed improvements in physical fitness variables regardless of their participation in the intervention, while in females, the accumulation of adiposity caused by puberty could not be mitigated by the intervention with mobile apps, as was the case in males. These results underscore the differential interaction between biological maturation, gender, and behavioral interventions during adolescence, and highlight the importance of considering gender-specific mechanisms when designing and evaluating mobile-app-based physical activity interventions. Although gender-related physiological differences may contribute to these findings, the present study did not directly evaluate hormonal, endocrine or neuromuscular mechanisms; therefore, these explanations should be interpreted as plausible rather than definitive. Furthermore, it is critical to acknowledge that the observed significant higher-order interactions for gender-related adaptations yielded generally small to trivial effect sizes. Consequently, while statistically sound, the absolute clinical or functional significance of these differences remains modest, and the recommendation to design gender-tailored interventions should be treated as a cautious hypothesis for future investigation rather than an immediate practical directive.

From a practical standpoint, these findings suggest that mobile technology interventions can be applied across diverse adolescent cohorts without requiring substantial or complex structural adaptations, as evidenced by the absence of widespread maturation-related differences in physical activity levels. While the interaction analyses indicate that maturity status and gender may play an outcome-specific role, particularly when targeting adiposity-related variables, the small-to-trivial associated effect sizes mean these variations should be interpreted as subtle phenotypic nuances rather than rigid directives for practice. Consequently, the advantage typically associated with early biological maturation in competitive sport contexts does not seem to be as decisive in mobile-based interventions implemented within educational settings. This highlights the substantial potential of school-based mHealth programs to promote physical activity inclusively, allowing adolescents at disparate stages of biological maturation and different genders to effectively participate in the same tracking regimen without the need for resource-intensive personalization.

Despite the relevant findings, this study has some limitations that should be considered. First, the intervention was based on the use of mobile step-tracking apps, but the intensity with which participants completed the program was not considered. Second, although a large sample of adolescents was used, convenience sampling and the fact that the study was conducted in only two schools may limit the generalizability of the results to other sociocultural or geographic contexts. Third, physical activity levels were assessed using a self-administered questionnaire. Although this instrument has demonstrated acceptable validity, self-reported measures are inherently susceptible to recall errors and social desirability bias, which may have led to overestimations of habitual active time. Fourth, biological maturity was estimated using the maturity offset equation rather than direct assessment methods such as skeletal age. Although this non-invasive method has been widely validated and is commonly used in adolescent research, it remains an indirect estimate that carries inherent mathematical error margins, which may be less accurate in individuals with very early or very late maturation. Fifth, a final statistical consideration must be made regarding the large number of dependent variables and multi-factor interactions analyzed, which inherently increases the probability of Type I errors. While a strict Bonferroni correction was systematically applied across all post hoc comparisons to mitigate this risk, the presence of marginal significance alongside small effect sizes means that a potential residual Type I error cannot be entirely dismissed, necessitating a prudent interpretation of the specific higher-order interactions. Future studies should incorporate objective measures of physical activity, such as accelerometry, together with more direct assessments of biological maturation to further clarify the role of maturation in adolescents’ responses to mHealth interventions.

## 5. Conclusions

In conclusion, this study demonstrates that a 10-week mobile application-based step-tracking intervention can be effectively implemented in a school setting to support physical activity. Notably, biological maturity status does not act as a global or dominant determinant of the intervention’s success. Its moderating role was limited to specific, minor variations in adiposity variables among early-maturing males. Furthermore, although higher-order interactions involving biological maturation and gender achieved statistical significance for certain anthropometric and fitness parameters, their associated effect sizes were consistently small to trivial. From a practical standpoint, the modest magnitude of these interaction terms indicates limited functional relevance, suggesting that natural pubertal growth patterns largely eclipse or mask the short-term effects of digital tracking. Consequently, rather than supporting immediate directives for maturity-specific or gender-tailored digital platforms, these findings offer highly positive practical implications for physical education. Because the real-world impact of biological and gender differences on outcomes is minor, a standardized mobile step-tracking strategy can be applied collectively to diverse groups of students, ensuring inclusive participation within the same physical activity framework.

## Figures and Tables

**Table 1:** Intra-group difference (pre-post) in the intervention and control groups according to maturity status.

Variable	Intervention Group	Maturation Group	Pre-Test (M ± SD)	Post-Test (M ± SD)	Mean Diff. (Pre-Post)	Time × Group × Maturity Status			Time × Group × Maturity Status × Gender		
						F	*p*	$\eta_{p}^{2}$	F	*p*	$\eta_{p}^{2}$
Body mass (kg)	EG	Early	66.22 ± 16.76	67.56 ± 16.41	−1.34	0.042	0.959	0.000	2.970	0.046	0.001
		On-time	53.43 ± 9.47	54.31 ± 9.33	−0.89						
		Late	53.22 ± 12.00	53.99 ± 11.68	−0.77						
	CG	Early	61.82 ± 12.57	62.98 ± 13.13	−1.16						
		On-time	52.24 ± 9.74	53.13 ± 9.47	−0.89						
		Late	48.60 ± 8.50	49.46 ± 8.14	−0.86						
Height (cm)	EG	Early	172.07 ± 9.99	172.93 ± 9.70	−0.86	0.708	0.493	0.004	5.912	0.016	0.018
		On-time	163.74 ± 8.14	164.48 ± 8.11	−0.74						
		Late	158.02 ± 6.00	158.71 ± 5.81	−0.69						
	CG	Early	167.94 ± 9.99	168.61 ± 9.80	−0.67						
		On-time	161.11 ± 8.03	161.76 ± 8.11	−0.65						
		Late	157.39 ± 7.29	158.01 ± 7.05	−0.62						
Sum of 3 skinfolds (mm)	EG	Early	46.83 ± 22.85	43.67 ± 19.97	3.15	3.281	0.039	0.020	3.663	0.016	0.012
		On-time	45.02 ± 22.42	44.33 ± 20.66	0.69						
		Late	62.77 ± 29.92	60.15 ± 26.71	2.62						
	CG	Early	46.26 ± 33.56	47.73 ± 31.30	−1.47						
		On-time	42.85 ± 23.30	41.28 ± 22.23	1.58						
		Late	46.79 ± 19.13	46.87 ± 19.33	−0.09						
Waist girth (cm)	CG	Early	74.76 ± 11.02	74.98 ± 10.72	−0.22	0.871	0.419	0.005	0.900	0.049	0.012
		On-time	67.12 ± 6.81	67.03 ± 6.19	0.10						
		Late	67.53 ± 8.49	67.62 ± 8.25	−0.09						
	EG	Early	72.19 ± 8.18	73.29 ± 8.73	−1.10						
		On-time	67.83 ± 6.53	67.73 ± 6.43	0.10						
		Late	64.95 ± 6.12	65.36 ± 6.02	−0.41						
Fat mass (%)	EG	Early	21.53 ± 10.20	20.36 ± 9.09	1.16	2.350	0.037	0.014	3.427	0.014	0.010
		On-time	20.02 ± 8.81	19.71 ± 8.52	0.31						
		Late	26.58 ± 11.14	25.89 ± 10.36	0.69						
	CG	Early	20.98 ± 14.40	21.61 ± 13.90	−0.63						
		On-time	19.20 ± 9.86	18.58 ± 9.63	0.63						
		Late	20.68 ± 7.53	20.47 ± 7.43	0.22						
Handgrip right arm (kg)	EG	Early	31.08 ± 8.26	33.17 ± 9.56	−2.09	1.516	0.221	0.009	2.210	0.047	0.007
		On-time	24.74 ± 7.46	26.53 ± 7.96	−1.79						
		Late	21.42 ± 5.54	22.42 ± 5.52	−1.00						
	CG	Early	28.08 ± 8.58	28.53 ± 11.54	−0.46						
		On-time	25.27 ± 7.37	26.33 ± 8.37	−1.06						
		Late	21.48 ± 5.12	22.24 ± 6.07	−0.76						
Handgrip left arm (kg)	EG	Early	29.07 ± 7.37	30.40 ± 7.92	−1.33	0.945	0.390	0.006	2.352	0.033	0.009
		On-time	23.46 ± 7.10	24.28 ± 7.47	−0.82						
		Late	19.93 ± 5.00	20.62 ± 4.75	−0.69						
	CG	Early	26.14 ± 7.05	27.83 ± 8.08	−1.69						
		On-time	24.09 ± 7.34	24.73 ± 8.44	−0.64						
		Late	20.64 ± 4.74	20.90 ± 6.03	−0.26						
Curl-up (reps)	EG	Early	21.67 ± 9.51	25.69 ± 12.68	−4.03	0.125	0.882	0.001	3.963	0.006	0.013
		On-time	22.30 ± 12.61	25.28 ± 10.76	−2.98						
		Late	17.97 ± 10.48	22.82 ± 9.61	−4.85						
	CG	Early	23.17 ± 9.30	23.92 ± 8.95	−0.75						
		On-time	21.89 ± 10.78	23.43 ± 12.65	−1.54						
		Late	16.35 ± 12.15	18.75 ± 11.24	−2.40						

EG: experimental group; CG: control group.

## Data Availability

The data that support the findings of this study are available on https://doi.org/10.5281/zenodo.14901853.

## Supplementary Materials

The following supporting information can be downloaded at: https://www.mdpi.com/article/10.3390/sports14080317/s1, Table S1: Intra-group difference (pre-post) in the intervention and control groups according to maturity status; Table S2: Intra-group difference (pre-post) in the intervention and control groups according to the stage of maturation in males; Table S3: Intra-group difference (pre-post) in the intervention and control groups according to the stage of maturation in females.
