# Supplementary material for: Influence of Maturity Status and Gender on Body Composition and Physical Fitness Adaptations to a Mobile-App-Based Physical Activity Intervention in Adolescents
Source: Sports (Basel). 2026 Jul 24;14(8):317. doi: 10.3390/sports14080317 (PMC13517363; doi:10.3390/sports14080317)
Supplement: Supplementary file 1 [file sports-14-00317-s001.zip › sports-4412141-supplementary.pdf]

The Bonferroni post hoc analysis for the three-way (Time × Group × Maturity Status) interaction model revealed differences in most of the maturation groups, with no clear differences in one of them. In the EG, there was a significant increase in all maturation groups in body mass (p<0.001), height (p<0.001-0.001), corrected arm girth (p<0.001-0.011), corrected thigh girth (p<0.001-0.037), sum of corrected girths (p<0.001), hip girth (p<0.001-0.007), muscle mass (p<0.001), right arm handgrip (p<0.001-0.035), push-ups (p=0.003-0.040) and curl-ups (p<0.001-0.020). A significant increase was also found in the early and on-time maturers in the variables BMI (p=0.02-p=0.039), corrected calf girth (p=0.026-0.044), left arm handgrip (p=0.020-0.021); for on-time and late maturers in the variables physical activity level (p=<0.001-0.004) and VO_2 max. (p<0.001); and for on-time maturers in CMJ (p=0.002). The early and late maturer EG participants showed a significant decrease in the sum of 3 skinfolds (p=0.006-0.029), and the early maturers group also in the fat mass variable (p=0.033).

In the CG, there was an increase in all maturation groups in body mass (p<0.001-0.003), height (p<0.001-0.047), corrected arm girth (p=0.001-0.017), sum of corrected girths (p<0.001-0.028), hip girth (p<0.001), and muscle mass (p=0.002-0.025). There was also an increase in BMI (p=0.017-0.042) in the early and on-time maturation groups; for the on-time and late groups, in corrected calf girth (p=0.027-0.043); for the early group, in the waist girth variable (p=0.017) and left arm handgrip (p=0.021); for the on-time group, in the VO_2 max (p=0.005), right arm handgrip (p=0.038), and push-up (p=0.012) variables; and in the late group, in the corrected thigh girth variable (p=0.019).

Supplementary Table S2 shows the differences in the study variables between the pre- and post-test measurements in males. In the EG, there was a significant increase in the on-time maturation group in body mass (p<0.001), BMI (p=0.002), corrected arm girth (p<0.001), corrected thigh girth (p=0.002), sum of corrected girths (p<0.001), hip girth (p<0.001), muscle mass (p<0.001), VO_2 max. (p<0.001), right arm handgrip (p<0.001), CMJ (p<0.001), and push-ups (p=0.002). In addition, the early group showed significant increases in the sum of corrected girths (p=0.017), muscle mass (p=0.008), left arm handgrip (p=0.041), and curl-ups (p=0.044), while the late group showed a significant increase in VO_2 max. (p=0.026), push-ups (p=0.030), and curl-ups (p=0.024). The height variable increased significantly in all maturation groups (p<0.001-0.011). The sum of 3 skinfolds (p=0.005) and fat mass (p=0.002) decreased after the intervention in the early group.

In the CG, there was a significant increase in the on-time and late groups in body mass (p<0.001-0.025), height (p<0.001-0.002), corrected arm girth (p=0.001-0.009), sum of corrected girths (p=0.010-0.011), hip girth (p<0.001-0.006), muscle mass (p=0.017-0.019), and VO_2 max. (p=0.005-0.015). In addition, in the on-time group, there were increases in BMI (p=0.027), corrected calf girth (p=0.043), right arm handgrip (p=0.012), left arm handgrip (p=0.007), push-ups (p=0.023), and curl-ups (p=0.013).

The differences in the study variables between the pre- and post-test measurements in females are shown in Supplementary Table S3. In the EG, a significant increase was found in all three maturation groups in the variables sum of corrected girths (p<0.001-0.005), muscle mass (p<0.001-0.025), and curl-up (p=0.001-0.042). A significant increase was also found in the early and on-time groups, in the variables body mass (p<0.001-0.019), corrected thigh girth (p<0.001-0.003), and hip girth (p<0.001-0.002); for the on-time and late groups in the variables physical activity level (p=0.002-0.036) and corrected arm girth (p<0.001-0.022); and in the on-time group in the variables height (p<0.001), right arm handgrip (p=0.009), and left arm handgrip (p=0.031).

In the CG, there was an increase in the on-time group in body mass (p<0.001), height (p=0.037), BMI (p<0.001), corrected arm girth (p=0.002), corrected thigh girth (p=0.019), sum of corrected girths (p<0.001), hip girth (p<0.001), and muscle mass (p=0.003), as well as in the early group in height (p<0.001) and BMI (p=0.007).

Supplementary Table S1. Intra-group difference (pre-post) in the intervention and control groups according to maturity status.

Variable	Intervention group	Maturation group	Pre-test (M±SD)	Post-test (M±SD)	Mean Diff. (pre-post)	Time*Group*Maturity Status			Time*Group*Maturity Status*Gender		
						F	p	ηp2	F	p	ηp2
Physical activity level	EG	Early	2.77±0.53	2.90±0.58	-0.13	0.049	0.952	0.000	2.426	0.120	0.007
		On-time	2.77±0.63	2.93±0.54	-0.16						
		Late	2.41±0.71	2.61±0.62	-0.20						
	CG	Early	2.97±0.59	2.95±0.52	0.02						
		On-time	2.81±0.58	2.75±0.75	0.06						
		Late	2.49±0.78	2.54±0.79	-0.06						
Body mass (kg)	EG	Early	66.22±16.76	67.56±16.41	-1.34	0.042	0.959	0.000	2.970	0.046	0.001
		On-time	53.43±9.47	54.31±9.33	-0.89						
		Late	53.22±12.00	53.99±11.68	-0.77						
	CG	Early	61.82±12.57	62.98±13.13	-1.16						
		On-time	52.24±9.74	53.13±9.47	-0.89						
		Late	48.60±8.50	49.46±8.14	-0.86						
Height (cm)	EG	Early	172.07±9.99	172.93±9.70	-0.86	0.708	0.493	0.004	5.912	0.016	0.018
		On-time	163.74±8.14	164.48±8.11	-0.74						
		Late	158.02±6.00	158.71±5.81	-0.69						
	CG	Early	167.94±9.99	168.61±9.80	-0.67						
		On-time	161.11±8.03	161.76±8.11	-0.65						
		Late	157.39±7.29	158.01±7.05	-0.62						
BMI (kg/m^2)	EG	Early	22.28±4.34	22.55±4.33	-0.27	0.136	0.873	0.001	0.245	0.621	0.001
		On-time	18.89±2.81	20.04±2.66	-0.15						
		Late	21.35±4.34	21.38±4.15	-0.04						

 https://doi.org/10.3390/xxxxx

	CG	Early	21.99±3.94	22.29±3.87	-0.30						
		On-time	20.07±3.14	20.26±2.99	-0.20						
		Late	19.55±3.11	19.62±2.99	-0.07						
Sum of 3 skinfolds (mm)	EG	Early	46.83±22.85	43.67±19.97	3.15	3.281	0.039	0.020	3.663	0.016	0.012
		On-time	45.02±22.42	44.33±20.66	0.69						
		Late	62.77±29.92	60.15±26.71	2.62						
	CG	Early	46.26±33.56	47.73±31.30	-1.47						
		On-time	42.85±23.30	41.28±22.23	1.58						
		Late	46.79±19.13	46.87±19.33	-0.09						
Corrected arm girth (cm)	EG	Early	23.61±3.38	24.01±3.20	-0.40	0.289	0.749	0.002	0.677	0.411	0.002
		On-time	20.72±2.48	21.10±2.35	-0.38						
		Late	19.97±2.05	20.42±2.05	-0.45						
	CG	Early	22.27±2.82	22.75±2.68	-0.49						
		On-time	21.11±2.63	21.44±2.59	-0.34						
		Late	19.58±2.26	20.01±2.12	-0.44						
Corrected thigh girth (cm)	EG	Early	43.50±4.84	44.25±5.02	-0.76	0.485	0.616	0.003	0.074	0.786	0.000
		On-time	39.03±4.28	39.87±4.07	-0.84						
		Late	37.60±3.87	38.74±3.83	-1.14						
	CG	Early	42.36±3.95	42.90±3.53	-0.53						
		On-time	39.78±5.76	40.03±4.33	-0.25						
		Late	37.11±3.49	37.85±3.13	-0.74						
Corrected calf girth (cm)	EG	Early	31.38±2.62	31.99±2.88	-0.61	0.282	0.754	0.002	1.505	0.221	0.005
		On-time	29.98±2.86	29.40±2.77	-0.42						
		Late	27.91±3.88	28.11±2.05	-0.20						
	CG	Early	30.23±3.08	30.72±2.84	-0.49						
		On-time	29.01±2.65	29.49±2.62	-0.48						
		Late	27.62±2.12	28.15±2.02	-0.53						
	EG	Early	98.49±9.75	100.26±10.27	-1.77	0.683	0.506	0.004	0.615	0.434	0.002

		On-time	88.74±8.91	90.37±8.61	-1.63						
Sum of corrected girths (cm)		Late	85.48±8.23	87.27±7.11	-1.79						
	CG	Early	94.86±8.75	96.37±8.00	-1.51						
		On-time	89.90±9.80	90.96±8.93	-1.07						
		Late	84.31±7.11	86.02±6.53	-1.71						
Waist girth (cm)	CG	Early	74.76±11.02	74.98±10.72	-0.22						
		On-time	67.12±6.81	67.03±6.19	0.10						
		Late	67.53±8.49	67.62±8.25	-0.09	0.871	0.419	0.005	0.900	0.049	0.012
	EG	Early	72.19±8.18	73.29±8.73	-1.10						
		On-time	67.83±6.53	67.73±6.43	0.10						
		Late	64.95±6.12	65.36±6.02	-0.41						
Hip girth (cm)	CG	Early	93.18±9.97	94.17±9.68	-0.99						
		On-time	87.51±7.23	88.52±6.78	-1.01						
		Late	90.13±10.13	90.95±9.54	-0.82	0.760	0.469	0.005	2.773	0.097	0.008
	EG	Early	90.41±7.62	92.09±7.92	-1.69						
		On-time	85.75±7.68	86.66±7.55	-0.91						
		Late	84.99±7.35	86.71±7.02	-1.72						
Waist/height ratio	EG	Early	0.44±0.07	0.43±0.06	0.00						
		On-time	0.41±0.04	0.41±0.03	0.00						
		Late	0.43±0.05	0.43±0.05	0.00	0.456	0.634	0.003	0.863	0.354	0.003
	CG	Early	0.43±0.06	0.44±0.06	-0.00						
		On-time	0.42±0.04	0.42±0.04	0.00						
		Late	0.41±0.04	0.41±0.04	-0.00						
Muscle mass (kg)	EG	Early	23.80±5.28	24.60±5.63	-0.80						
		On-time	18.03±4.49	18.68±4.47	-0.65						
		Late	15.56±3.00	16.26±2.84	-0.71	0.507	0.602	0.003	0.128	0.721	0.000
	CG	Early	21.91±4.25	22.53±3.97	-0.62						
		On-time	19.04±4.58	19.39±4.25	-0.35						

		Late	15.46±3.21	16.05±3.05	-0.59						
Fat mass (%)	EG	Early	21.53±10.20	20.36±9.09	1.16	2.350	0.037	0.014	3.427	0.014	0.010
		On-time	20.02±8.81	19.71±8.52	0.31						
		Late	26.58±11.14	25.89±10.36	0.69						
	CG	Early	20.98±14.40	21.61±13.90	-0.63						
		On-time	19.20±9.86	18.58±9.63	0.63						
		Late	20.68±7.53	20.47±7.43	0.22						
VO_2 max. (ml/kg/min)	EG	Early	39.93±4.57	40.76±5.20	-0.83	0.343	0.710	0.002	0.070	0.791	0.000
		On-time	39.43±4.54	40.92±5.52	-1.49						
		Late	35.56±4.40	36.68±4.97	-1.12						
	CG	Early	41.05±5.31	40.90±4.00	0.14						
		On-time	39.29±4.78	40.30±5.30	-1.00						
		Late	36.54±4.58	37.06±4.65	-0.52						
Handgrip right arm (kg)	EG	Early	31.08±8.26	33.17±9.56	-2.09	1.516	0.221	0.009	2.210	0.047	0.007
		On-time	24.74±7.46	26.53±7.96	-1.79						
		Late	21.42±5.54	22.42±5.52	-1.00						
	CG	Early	28.08±8.58	28.53±11.54	-0.46						
		On-time	25.27±7.37	26.33±8.37	-1.06						
		Late	21.48±5.12	22.24±6.07	-0.76						
Handgrip left arm (kg)	EG	Early	29.07±7.37	30.40±7.92	-1.33	0.945	0.390	0.006	2.352	0.033	0.009
		On-time	23.46±7.10	24.28±7.47	-0.82						
		Late	19.93±5.00	20.62±4.75	-0.69						
	CG	Early	26.14±7.05	27.83±8.08	-1.69						
		On-time	24.09±7.34	24.73±8.44	-0.64						
		Late	20.64±4.74	20.90±6.03	-0.26						
CMJ (cm)	EG	Early	25.60±7.06	25.88±8.06	-0.28	0.069	0.933	0.000	1.690	0.194	0.005
		On-time	22.57±7.53	24.98±7.12	-2.41						
		Late	20.28±5.94	20.44±7.77	-0.15						

	CG	Early	22.38±9.04	23.22±8.93	-0.83						
		On-time	23.46±6.87	23.88±9.55	-0.42						
		Late	20.68±6.57	20.68±8.91	0.01						
Push-ups (reps)	EG	Early	10.55±12.98	12.66±12.19	-2.11	0.619	0.539	0.004	0.018	0.895	0.000
		On-time	7.50±9.12	9.35±11.42	-1.85						
		Late	4.10±6.89	5.55±7.24	-1.45						
	CG	Early	8.32±9.66	7.86±11.57	0.45						
		On-time	9.44±9.95	11.26±10.50	-1.82						
		Late	5.35±7.73	6.00±6.92	-0.65						
Curl-up (reps)	EG	Early	21.67±9.51	25.69±12.68	-4.03	0.125	0.882	0.001	3.963	0.006	0.013
		On-time	22.30±12.61	25.28±10.76	-2.98						
		Late	17.97±10.48	22.82±9.61	-4.85						
	CG	Early	23.17±9.30	23.92±8.95	-0.75						
		On-time	21.89±10.78	23.43±12.65	-1.54						
		Late	16.35±12.15	18.75±11.24	-2.40						

EG: experimental group; CG: control group; BMI: body mass index; VO2 max.: maximum oxygen consumption; CMJ: countermovement jump.

sports

MDPI

Supplementary Table S2. Intra-group difference (pre-post) in the intervention and control groups according to the stage of maturation in males.

Variable	Intervention group	Maturation group	Pre-test (M±SD)	Post-test (M±SD)	Mean Diff. (pre-post)	p	95% CI	ηp2
Physical activity level	EG	Early	2.73±0.56	2.92±0.58	-0.19	0.111	-0.42; 0.04	0.007
		On-time	2.90±0.65	3.00±0.57	-0.10	0.153	-0.24; 0.04	0.005
		Late	2.59±0.56	2.85±0.44	-0.26	0.077	-0.55; 0.03	0.008
	CG	Early	3.06±0.36	3.05±0.48	0.01	0.959	-0.33; 0.34	0.000
		On-time	2.88±0.58	2.83±0.69	0.05	0.446	-0.08; 0.19	0.002
		Late	2.75±0.93	2.73±0.82	0.02	0.892	-0.21; 0.24	0.000
Body mass (kg)	EG	Early	72.00±18.71	72.63±18.73	-0.64	0.101	-1.40; 0.13	0.007
		On-time	55.66±9.40	57.12±9.48	-1.46	<0.001	-1.91; -1.01	0.098
		Late	47.96±8.79	48.80±8.69	-0.85	0.079	-1.79; 0.10	0.008
	CG	Early	65.69±15.64	66.65±16.65	-1.06	0.061	-2.16; 0.05	0.009
		On-time	53.70±9.42	54.70±8.96	-1.01	<0.001	-1.46; -0.56	0.048
		Late	47.04±7.94	47.88±7.32	-0.84	0.025	-1.57; -0.10	0.013
Height (cm)	EG	Early	175.01±7.50	175.85±7.36	-0.84	0.011	-1.49; -0.19	0.017
		On-time	166.88±9.66	167.89±9.39	-1.01	<0.001	-1.39; -0.63	0.067
		Late	158.63±7.53	159.98±7.48	-1.35	0.001	-2.16; -0.55	0.028
	CG	Early	168.64±10.17	169.22±9.75	-0.58	0.224	-1.52; 0.36	0.004
		On-time	162.78±9.19	163.75±9.09	-0.97	<0.001	-1.36; -0.59	0.061
		Late	157.90±7.11	158.91±6.88	-1.01	0.002	-1.64; -0.39	0.026
BMI (kg/m²)	EG	Early	23.38±4.84	23.42±4.94	-0.04	0.794	-0.33; 0.26	0.008
		On-time	19.97±2.60	20.24±2.66	-0.27	0.002	-0.44; -0.10	0.000
		Late	18.98±2.79	19.02±2.55	-0.03	0.857	-0.40; 0.33	0.002
	CG	Early	23.05±4.59	23.27±4.38	-0.22	0.303	-0.65; 0.20	0.019

		On-time	20.23±3.02	20.43±2.84	-0.20	0.027	-0.37; -0.02	0.031
		Late	18.88±2.83	18.97±2.56	-0.09	0.540	-0.37; 0.19	0.005
Sum of 3 skinfolds (mm)	EG	Early	48.54±23.81	43.26±19.20	5.28	0.005	1.58; 8.98	0.021
		On-time	37.49±19.60	36.66±18.57	0.84	0.448	-1.33; 3.00	0.002
		Late	37.89±20.19	37.46±21.90	0.43	0.854	-4.15; 5.01	0.000
	CG	Early	54.49±37.68	51.87±33.20	2.62	0.336	-2.73; 7.97	0.002
		On-time	39.04±22.66	38.60±21.98	0.44	0.695	-1.75; 2.62	0.000
		Late	35.28±16.97	32.37±14.87	2.91	0.122	-0.79; 6.61	0.006
Corrected arm girth (cm)	EG	Early	24.44±3.36	24.66±2.98	-0.22	0.282	-0.62; 0.18	0.003
		On-time	21.96±2.56	22.42±2.54	-0.45	<0.001	-0.69; -0.22	0.038
		Late	19.66±1.79	20.09±1.83	-0.43	0.085	-0.93; 0.06	0.008
	CG	Early	22.78±3.45	22.83±3.17	-0.05	0.872	-0.62; 0.53	0.000
		On-time	21.54±2.65	21.93±2.65	-0.39	0.001	-0.62; -0.15	0.027
		Late	20.32±2.37	20.85±2.34	-0.53	0.009	-0.93; -0.14	0.018
Corrected thigh girth (cm)	EG	Early	45.35±4.30	46.07±4.86	-0.72	0.130	-1.65; 0.21	0.006
		On-time	40.91±3.96	41.77±3.72	-0.86	0.002	-1.40; -0.31	0.025
		Late	37.66±3.54	38.21±4.00	-0.56	0.342	-1.71; 0.60	0.002
	CG	Early	43.11±4.81	43.85±4.23	-0.73	0.285	-2.08; 0.61	0.003
		On-time	40.79±5.83	40.98±4.19	-0.19	0.501	-0.74; 0.36	0.001
		Late	37.77±3.83	38.51±3.20	-0.74	0.119	-1.67; 0.19	0.006
Corrected calf girth (cm)	EG	Early	32.45±2.58	33.16±2.87	-0.71	0.073	-1.49; 0.07	0.009
		On-time	30.36±2.46	30.74±2.52	-0.38	0.099	-0.84; 0.07	0.007
		Late	27.89±2.35	28.37±2.54	-0.48	0.323	-1.45; 0.48	0.003
	CG	Early	30.82±3.87	31.46±3.37	-0.64	0.265	-1.76; 0.49	0.003
		On-time	29.53±2.68	30.00±2.63	-0.47	0.043	-0.93; -0.01	0.011
		Late	28.12±2.16	28.60±2.11	-0.48	0.226	-1.26; 0.30	0.004
Sum of corrected girths (cm)	EG	Early	102.24±8.62	103.89±9.88	-1.65	0.017	-3.00; -0.30	0.015
		On-time	93.23±8.43	94.93±8.05	-1.69	<0.001	-2.49; -0.90	0.045

		Late	85.20±7.30	86.68±8.00	-1.48	0.084	-3.15; 0.20	0.008
	CG	Early	96.72±10.92	98.14±9.73	-1.42	0.154	-3.37; 0.54	0.005
		On-time	91.86±9.83	92.91±8.84	-1.05	0.010	-1.85; -0.25	0.018
		Late	86.21±7.68	87.96±6.93	-1.75	0.011	-3.10; -0.40	0.017
Waist girth (cm)	EG	Early	77.45±11.83	77.35±12.06	0.10	0.826	-0.81; 1.01	0.000
		On-time	69.49±7.13	69.44±6.63	0.06	0.830	-0.48; 0.59	0.000
		Late	66.70±6.15	67.01±5.74	-0.31	0.590	-1.44; 0.82	0.001
	CG	Early	75.64±9.79	75.84±10.37	-0.20	0.769	-1.51; 1.12	0.000
		On-time	68.61±6.26	69.06±6.37	-0.46	0.096	-0.99; 0.08	0.007
		Late	66.83±5.10	66.61±5.22	0.22	0.632	-0.69; 1.13	0.001
Hip girth (cm)	EG	Early	96.13±10.95	96.48±10.95	-0.35	0.461	-1.28; 0.58	0.001
		On-time	87.31±7.24	88.30±6.80	-0.99	<0.001	-1.53; -0.44	0.033
		Late	82.88±7.23	83.92±7.22	-1.04	0.077	-2.19; 0.11	0.008
	CG	Early	93.55±9.30	94.54±9.55	-0.99	0.150	-2.33; 0.36	0.006
		On-time	85.65±7.05	86.75±7.01	-1.10	<0.001	-1.65; -0.55	0.040
		Late	81.39±5.57	82.70±4.84	-1.31	0.006	-2.24; -0.38	0.020
Waist/height ratio	EG	Early	0.44±0.06	0.44±0.06	0.003	0.363	-0.00; 0.01	0.002
		On-time	0.42±0.05	0.41±0.04	0.003	0.076	0.00; 0.01	0.008
		Late	0.42±0.04	0.42±0.03	0.002	0.620	-0.01; 0.01	0.001
	CG	Early	0.45±0.06	0.45±0.06	0.001	0.814	-0.01; 0.01	0.000
		On-time	0.42±0.04	0.42±0.04	0.000	0.837	-0.00; 0.00	0.000
		Late	0.42±0.03	0.42±0.03	0.004	0.149	-0.00; 0.10	0.006
Muscle mass (kg)	EG	Early	25.77±4.78	26.52±5.40	-0.75	0.008	-1.29; -0.20	0.019
		On-time	21.15±3.85	21.92±3.78	-0.77	<0.001	-1.09; -0.45	0.056
		Late	17.66±2.81	18.29±3.13	-0.63	0.068	-1.31; 0.05	0.009
	CG	Early	22.80±5.40	23.29±4.95	-0.49	0.227	-1.28; 0.30	0.004
		On-time	20.39±4.38	20.77±3.96	-0.39	0.019	-0.71; -0.06	0.015
		Late	18.03±3.09	18.70±2.91	-0.67	0.017	-1.21; -0.12	0.015

https://doi.org/10.3390/xxxxx

Fat mass (%)	EG	Early	22.63±10.86	20.43±8.99	2.20	0.002	0.81; 3.59	0.025
		On-time	17.08±8.21	16.78±8.31	0.31	0.457	-0.51; 1.12	0.001
		Late	17.19±8.14	16.71±8.82	0.48	0.582	-1.24; 2.21	0.001
	CG	Early	24.66±16.61	23.72±15.30	0.93	0.363	-1.08; 2.94	0.002
		On-time	17.67±9.63	17.53±9.73	0.15	0.727	-0.68; 0.97	0.000
		Late	16.19±7.69	14.98±6.81	1.21	0.088	-0.18; 2.60	0.008
VO_2 max. (ml/kg/min)	EG	Early	39.39±4.73	40.20±5.53	-0.81	0.221	-2.10; 0.49	0.005
		On-time	41.33±5.19	43.48±5.69	-2.15	<0.001	-2.87; -1.42	0.092
		Late	40.75±4.39	42.40±4.07	-1.66	0.026	-3.11; -0.20	0.015
	CG	Early	41.53±5.17	40.73±3.67	0.82	0.394	-1.06; 2.69	0.002
		On-time	40.41±4.82	41.51±4.77	-1.09	0.005	-1.85; -0.34	0.024
		Late	39.31±6.14	40.80±6.06	-1.49	0.015	-2.69; -0.29	0.018
Handgrip right arm (kg)	EG	Early	33.67±7.53	35.25±8.79	-1.58	0.095	-3.43; 0.27	0.007
		On-time	28.41±8.30	30.83±8.93	-2.42	<0.001	-3.50; -1.33	0.048
		Late	22.70±5.74	23.21±6.05	-0.51	0.664	-2.80; 1.79	0.000
	CG	Early	28.18±10.34	30.25±10.18	-2.06	0.131	-4.74; 0.62	0.006
		On-time	26.49±7.77	27.90±9.26	-1.40	0.012	-2.49; -0.32	0.017
		Late	23.46±7.13	24.40±6.97	-0.94	0.299	-2.72; 0.84	0.003
Handgrip left arm (kg)	EG	Early	31.13±7.10	32.67±8.21	-1.54	0.041	-3.01; -0.06	0.011
		On-time	27.27±7.15	28.00±7.63	-0.73	0.097	-1.59; 0.13	0.007
		Late	20.98±4.55	21.57±4.79	-0.59	0.527	-2.41; 1.24	0.001
	CG	Early	26.39±7.60	27.85±7.70	-1.46	0.180	-3.58; 0.67	0.005
		On-time	25.22±7.73	26.41±9.04	-1.19	0.007	-2.05; -0.33	0.019
		Late	22.24±6.24	22.85±7.00	-0.61	0.395	-2.02; 0.80	0.002
CMJ (cm)	EG	Early	25.85±7.06	25.29±8.35	0.56	0.730	-2.64; 3.76	0.000
		On-time	25.21±8.45	28.63±6.78	-3.42	<0.001	-5.30; -1.55	0.033
		Late	25.19±4.62	27.95±6.28	-2.76	0.171	-6.72; 1.20	0.005
	CG	Early	22.05±5.13	23.01±9.18	-0.96	0.685	-5.58; 3.67	0.000

		On-time	24.37±7.91	24.38±9.95	-0.01	0.995	-1.88; 1.87	0.000
		Late	24.03±7.07	25.04±9.86	-1.01	0.517	-4.08; 2.05	0.001
Push-ups (reps)	EG	Early	9.82±11.68	12.36±12.40	-2.55	0.058	-5.18; 0.09	0.010
		On-time	13.12±10.91	15.55±12.12	-2.43	0.002	-3.94; -0.92	0.027
		Late	7.87±6.64	11.40±6.91	-3.53	0.030	-6.72; -0.34	0.013
	CG	Early	8.73±8.43	7.45±8.37	1.27	0.502	-2.45; 5.00	0.001
		On-time	10.83±10.48	12.63±11.38	-1.81	0.023	-3.37; -0.25	0.014
		Late	11.38±9.71	10.25±9.27	1.13	0.381	-1.40; 3.65	0.002
Curl-up (reps)	EG	Early	23.83±9.07	28.35±9.91	-4.52	0.044	-8.9; -0.13	0.011
		On-time	25.78±12.73	27.66±12.29	-1.88	0.152	-4.45; 0.69	0.005
		Late	20.47±8.85	26.73±10.44	-6.27	0.024	-11.71; -0.83	0.013
	CG	Early	23.36±11.69	21.18±9.35	2.18	0.500	-4.17; 8.53	0.001
		On-time	22.53±9.48	25.80±11.65	-3.27	0.013	-5.87; -0.68	0.016
		Late	21.13±14.26	19.67±11.53	1.46	0.505	-2.84; 5.76	0.001

EG: experimental group; CG: control group; BMI: body mass index; VO2 max.: maximum oxygen consumption; CMJ: countermovement jump.

https://doi.org/10.3390/xxxxx

Supplementary Table S3. Intra-group difference (pre-post) in the intervention and control groups according to the stage of maturation in females.

Variable	Intervention group	Maturation group	Pre-test (M±SD)	Post-test (M±SD)	Mean Diff. (pre-post)	p	95% CI	ηp2
Physical activity level	EG	Early	2.65±0.40	2.88±0.49	-0.23	0.051	-0.46; 0.00	0.010
		On-time	2.54±0.64	2.73±0.54	-0.19	0.002	-0.31; -0.07	0.024
		Late	2.17±0.85	2.34±0.76	-0.17	0.036	-0.38; 0.05	0.016
	CG	Early	2.48±0.66	2.63±0.83	-0.15	0.452	-0.54; 0.24	0.001
		On-time	2.45±0.54	2.53±0.69	-0.09	0.323	-0.26; 0.09	0.003
		Late	2.48±1.07	2.11±1.22	0.37	0.084	-0.05; 0.79	0.008
Body mass (kg)	EG	Early	57.21±9.46	58.13±9.19	-0.91	0.019	-1.68; -0.15	0.014
		On-time	51.63±11.87	52.35±11.45	-0.73	<0.001	-1.14; -0.32	0.032
		Late	55.12±9.21	55.52±8.87	-0.40	0.271	-1.12; 0.32	0.003
	CG	Early	58.71±11.22	59.21±12.72	-0.50	0.449	-1.80; 0.80	0.002
		On-time	48.75±8.83	49.91±8.66	-1.17	<0.001	-1.75; -0.59	0.040
		Late	54.18±9.21	53.16±10.34	1.03	0.179	-0.47; 2.52	0.005
Height (cm)	EG	Early	163.52±6.95	163.85±6.77	-0.34	0.308	-0.99; 0.31	0.003
		On-time	158.58±5.60	159.19±5.32	-0.61	<0.001	-0.95; -0.26	0.030
		Late	157.39±5.21	157.75±5.23	-0.36	0.250	-0.25; 0.97	0.003
	CG	Early	163.56±6.10	164.70±8.38	-1.14	<0.001	-2.23; -1.04	0.037
		On-time	157.92±7.24	158.45±7.02	-0.97	0.037	-1.02; -0.03	0.011
		Late	154.72±5.10	154.82±4.93	-1.01	0.867	-1.38; 1.16	0.000
BMI (kg/m²)	EG	Early	21.41±3.29	21.67±2.99	-0.26	0.079	-0.56; 0.03	0.000
		On-time	20.58±4.29	20.60±4.01	-0.01	0.889	-0.17; 0.15	0.024
		Late	22.30±3.67	22.40±3.56	-0.11	0.449	-0.38; 0.17	0.000
	CG	Early	21.87±3.26	22.56±3.20	-0.69	0.007	-1.19; -0.19	0.003

		On-time	19.42±2.79	19.81±2.69	-0.40	<0.001	-0.62; -0.18	0.013
		Late	22.87±5.15	22.47±5.80	0.40	0.172	-0.18; 0.98	0.001
Sum of 3 skin-folds (mm)	EG	Early	63.80±24.15	62.66±21.91	1.14	0.544	-2.56; 4.84	0.001
		On-time	58.86±27.74	56.94±23.47	1.91	0.058	-0.07; 3.90	0.010
		Late	71.08±26.74	68.38±23.85	2.70	0.128	-0.78; 6.17	0.006
	CG	Early	60.91±20.16	65.85±19.53	-4.94	0.148	-11.64; 1.76	0.006
		On-time	50.38±18.75	50.72±18.06	-0.34	0.812	-3.18; 2.50	0.000
		Late	69.72±26.34	68.61±23.56	1.11	0.763	-6.13; 8.35	0.000
Corrected arm girth (cm)	EG	Early	20.47±2.31	20.67±2.26	-0.20	0.320	-0.60; 0.20	0.003
		On-time	19.51±2.04	19.99±2.03	-0.47	<0.001	-0.69; -0.26	0.048
		Late	20.49±1.95	20.93±1.75	-0.44	0.022	-0.81; -0.06	0.014
	CG	Early	20.42±1.66	20.46±1.69	-0.04	0.925	-0.76; 0.69	0.000
		On-time	19.28±2.25	19.78±2.15	-0.50	0.002	-0.80; -0.19	0.026
		Late	20.15±1.50	20.46±1.34	-0.31	0.435	-1.09; 0.47	0.002
Corrected thigh girth (cm)	EG	Early	38.08±4.66	39.49±3.59	-1.40	0.003	-2.34; -0.47	0.023
		On-time	36.90±3.80	37.99±3.98	-1.09	<0.001	-1.59; -0.59	0.047
		Late	38.47±3.36	39.19±2.82	-0.72	0.105	-1.60; 0.15	0.007
	CG	Early	39.38±4.19	39.28±4.53	0.10	0.910	-1.59; -1.79	0.000
		On-time	36.62±3.28	37.48±3.19	-0.86	0.019	-1.57; -0.14	0.015
		Late	38.04±2.91	37.98±2.13	0.06	0.947	-1.76; -1.89	0.000
Corrected calf girth (cm)	EG	Early	27.95±2.69	28.55±1.96	-0.60	0.130	-1.38; 0.18	0.006
		On-time	27.73±4.03	27.80±2.04	-0.07	0.740	-0.49; 0.35	0.000
		Late	27.62±2.04	28.26±1.75	-0.65	0.083	-1.38; 0.09	0.008
	CG	Early	27.95±1.22	27.79±1.30	0.15	0.830	-1.25; 1.56	0.000
		On-time	27.47±2.01	27.95±2.08	-0.49	0.108	-1.09; 0.11	0.007
		Late	27.32±2.61	28.74±1.43	-1.42	0.068	-2.94; 0.10	0.009
Sum of corrected girths (cm)	EG	Early	86.50±8.06	88.71±6.69	-2.21	0.001	-3.56; -0.85	0.015
		On-time	84.15±8.31	85.78±7.35	-1.63	<0.001	-2.36; -0.91	0.050

https://doi.org/10.3390/xxxxx

		Late	86.57±6.09	88.38±5.14	-1.81	0.005	-3.08; -0.54	0.020
	CG	Early	87.74±6.44	87.53±7.13	0.22	0.862	-2.23; 2.67	0.000
		On-time	83.37±6.71	85.21±6.61	-1.84	<0.001	-2.88; -0.80	0.032
		Late	85.51±4.90	87.18±3.06	-1.67	0.216	-4.31; 0.98	0.004
Waist girth (cm)	EG	Early	68.07±6.94	68.01±6.32	0.07	0.889	-0.85; 0.98	0.000
		On-time	65.82±8.56	66.01±8.24	-0.19	0.445	-0.68; 0.30	0.002
		Late	68.16±7.31	67.94±6.85	0.22	0.612	-0.64; 1.08	0.001
	CG	Early	68.54±6.58	68.10±6.61	0.44	0.604	-1.22; 2.09	0.001
		On-time	63.17±4.89	63.48±4.78	-0.31	0.384	-1.01; 0.39	0.002
		Late	70.01±11.26	70.50±11.01	-0.49	0.588	-2.28; 1.29	0.001
Hip girth (cm)	EG	Early	91.93±7.18	93.38±7.06	-1.45	0.002	-2.38; -0.52	0.024
		On-time	88.58±9.71	89.65±9.02	-1.07	<0.001	-1.57; -0.57	0.045
		Late	93.56±7.30	93.90±6.76	-0.34	0.443	-1.22; 0.53	0.002
	CG	Early	91.91±6.93	92.51±8.56	-0.60	0.485	-2.29; 1.09	0.001
		On-time	86.11±7.25	87.97±7.05	-1.86	<0.001	-2.57; -1.14	0.065
		Late	91.05±9.27	92.27±9.17	-1.22	0.190	-3.04; 0.61	0.005
Waist/height ratio	EG	Early	0.42±0.05	0.42±0.04	0.001	0.616	-0.00; 0.01	0.001
		On-time	0.42±0.05	0.42±0.05	0.000	0.806	-0.00; 0.00	0.000
		Late	0.43±0.05	0.43±0.05	0.002	0.373	-0.00; 0.01	0.002
	CG	Early	0.42±0.04	0.43±0.04	-0.004	0.468	-0.01; 0.01	0.001
		On-time	0.40±0.03	0.40±0.03	-0.001	0.732	-0.01; 0.00	0.000
		Late	0.45±0.09	0.46±0.09	-0.003	0.599	-0.01; 0.01	0.001
Muscle mass (kg)	EG	Early	15.84±3.21	16.58±2.74	-0.74	0.008	-1.29; -0.20	0.019
		On-time	14.65±2.92	15.31±2.85	-0.66	<0.001	-0.96; -0.37	0.050
		Late	15.70±2.15	16.29±1.87	-0.59	0.025	-1.10; -0.07	0.013
	CG	Early	16.14±2.80	15.96±3.13	0.18	0.721	-0.81; 1.17	0.000
		On-time	14.35±2.59	15.00±2.58	-0.65	0.003	-1.07; -0.23	0.024
		Late	15.04±1.35	15.35±1.05	-0.31	0.576	-1.38; 0.77	0.001

https://doi.org/10.3390/xxxxx

Fat mass (%)	EG	Early	27.32±9.05	27.24±8.49	0.09	0.905	-1.31; 1.48	0.000
		On-time	25.23±10.22	24.85±9.12	0.39	0.309	-0.36; 1.13	0.003
		Late	29.64±9.89	28.51±9.06	1.13	0.090	-0.18; 2.44	0.008
	CG	Early	27.31±9.13	28.82±8.96	-1.51	0.241	-4.03; 1.01	0.004
		On-time	21.97±6.94	22.05±6.83	-0.08	0.884	-1.15; 0.99	0.000
		Late	29.74±10.00	27.94±8.36	1.80	0.195	-0.92; 4.52	0.004
VO_2 max. (ml/kg/min)	EG	Early	37.51±2.20	37.46±2.97	0.05	0.937	-1.15; 1-25	0.000
		On-time	35.98±3.11	36.49±3.81	-0.51	0.116	-1.16; 0.13	0.007
		Late	32.51±3.77	33.28±4.69	-0.78	0.265	-2.14; 0.59	0.004
	CG	Early	35.80±2.23	34.82±2.47	0.98	0.366	-1.15; 3.11	0.002
		On-time	36.00±2.84	36.12±3.22	-0.13	0.793	-1.06; 0.81	0.000
		Late	32.08±2.41	33.44±1.40	-1.35	0.345	-4.17; 1.46	0.003
Handgrip right arm (kg)	EG	Early	22.05±4.17	22.81±4.51	-0.76	0.420	-2.61; 1.09	0.002
		On-time	20.90±4.56	22.21±4.47	-1.32	0.009	-2.30; -0.34	0.018
		Late	20.79±5.86	21.97±6.42	-1.18	0.185	-2.92; 0.57	0.005
	CG	Early	24.03±4.63	22.30±9.23	1.73	0.070	0.58; 3.87	0.008
		On-time	20.76±4.32	21.06±5.64	-0.30	0.671	-1.69; 1.09	0.000
		Late	23.16±2.94	25.31±2.47	-2.16	0.207	-5.52; 1.20	0.004
Handgrip left arm (kg)	EG	Early	21.23±4.41	22.34±4.70	-1.10	0.141	-2.58; 0.37	0.006
		On-time	19.19±4.31	20.04±3.94	-0.86	0.031	-1.64; -0.08	0.012
		Late	19.67±5.76	20.14±5.93	-0.47	0.502	-1.86; 0.91	0.001
	CG	Early	24.44±3.60	23.18±5.16	1.26	0.321	-1.23; .76	0.003
		On-time	19.75±4.18	19.76±5.60	-0.01	0.986	-1.11; 1.09	0.000
		Late	21.19±3.19	21.81±2.82	-0.63	0.643	-3.30; 2.04	0.001
CMJ (cm)	EG	Early	19.68±3.99	19.80±4.10	-0.12	0.940	-3.32; 3.07	0.000
		On-time	19.86±5.12	20.33±5.60	-0.47	0.585	-2.16; 1.22	0.001
		Late	18.93±7.25	18.12±10.00	0.82	0.594	-2.19; 3.82	0.001
	CG	Early	17.06±8.32	21.18±4.20	-4.11	0.136	-9.53; 1.31	0.006

		On-time	19.98±3.99	19.39±7.57	0.59	0.629	-1.81; 2.98	0.001
		Late	17.09±8.97	18.23±10.13	-1.14	0.698	-6.94; 4.65	0.000
Push-up (reps)	EG	Early	0.91±1.62	2.91±5.61	-2.00	0.128	-4.58; 0.58	0.006
		On-time	2.36±3.86	2.90±4.76	-0.54	0.445	-1.92; 0.84	0.002
		Late	5.13±10.03	6.79±9.05	-1.67	0.194	-4.19; 0.86	0.005
	CG	Early	0.43±0.79	3.00±4.55	-2.57	0.279	-7.24; 2.10	0.003
		On-time	2.79±4.30	4.26±6.08	-1.46	0.147	-3.44; 0.52	0.006
		Late	3.33±6.74	5.83±5.42	-2.50	0.330	-7.54; 2.54	0.003
Curl-up (reps)	EG	Early	16.09±9.31	20.65±9.99	-4.57	0.042	-8.96; -0.17	0.011
		On-time	17.04±9.64	20.73±8.21	-3.69	0.002	-6.03; -1.35	0.025
		Late	18.88±13.27	26.13±10.95	-7.25	0.001	-11.55; -2.95	0.028
	CG	Early	18.63±6.26	19.38±8.38	-0.75	0.843	-8.20; 6.70	0.000
		On-time	17.79±11.84	19.13±12.22	-1.33	0.437	-4.71; 2.04	0.002
		Late	5.86±4.71	13.00±10.08	-7.14	0.078	-15.10; 0.82	0.008

EG: experimental group; CG: control group; BMI: body mass index; VO2 max.: maximum oxygen consumption; CMJ: countermovement jump.
